# Food plants as adjuvant medicines: a review of protective effects and clinical potential in alcoholic liver disease

**DOI:** 10.3389/fphar.2025.1586238

**Published:** 2025-05-22

**Authors:** Chenyu Li, Qi Zhang, Zijun Chen, Weiming Hu, Fen Liu

**Affiliations:** ^1^ Basic Medical Experiment Center, School of Traditional Chinese Medicine, JiangXi University of Chinese Medicine, Nanchang, China; ^2^ Jiangxi Provincial Key Laboratory of Plant Germplasm Resources Innovation and Genetic Improvement, Lushan Botanical Garden, Jiangxi Province and Chinese Academy of Sciences, Jiujiang, China; ^3^ College of Life Sciences, Jiangxi Normal University, Nanchang, China; ^4^ Jiangxi Academy of Forestry, Nanchang, China

**Keywords:** alcoholic liver disease, food plants used as medicines, traditional Chinese Medicine, mechanism of action, pathogenesis of ALD

## Abstract

**Research background:**

Globally, alcohol usage is the third-leading risk factor for diseases, and alcohol-induced alcoholic liver disease (ALD) has become a global public health problem. ALD is characterized by oxidative stress and immune damage in the liver caused by excessive alcohol consumption. Furthermore, alcohol and its metabolites disrupt the health of the intestinal tract and cause secondary liver damage through the gut-liver axis.

**Existing problems:**

The underlying mechanisms of ALD are complex. Currently, there are no safe and effective drugs for the prevention and treatment of ALD; some food plants used as medicines (FPUM) have demonstrated promising effects in combating this condition.

**Solutions:**

In this review, we analyze the pathogenesis of ALD and explore the mechanisms of action of certain FPUM in preventing and treating ALD. Different mechanisms include activation of alcohol metabolism-related enzymes, maintenance of mitochondrial stability, reduction of oxidative stress damage caused by alcohol intake, regulation of cytokine levels, and modulation of the gut microbiota. The review also explores potential future research directions and summarizes insights for developing novel therapeutic agents and components.

**Future prospects:**

Future research on FPUM for the treatment of ALD could explore promising avenues such as multi-herb combinations, multi-component formulations, and side effect reduction strategies, demonstrating that the development of herbal medicine still holds boundless potential.

## 1 Introduction

With economic development and improvement in quality of life of humans, the proportion of people drinking alcohol and suffering from alcohol-related diseases has significantly increased. A recent report from the World Health Organization showed that 2.6 million people die every year as a result of alcohol consumption, accounting for 4.7% of all deaths (WHO, 2024). Alcohol consumption can cause damage to the liver, gastrointestinal tract, nervous system, cardiovascular system, and immune system, and chronic heavy alcohol consumption is a major risk factor for diseases such as fatty liver, alcoholic liver disease (ALD), cirrhosis, and liver cancer (Lieber, 1973; Massey et al., 2015; Natarajan et al., 2015; Kirpich et al., 2016).

ALD is a widespread liver abnormality caused by chronic or heavy alcohol consumption, characterized by liver damage, inflammation, fibrosis, cirrhosis, and cancer (Seitz et al., 2018). ALD often coexists with liver disease caused by viral hepatitis and non-alcoholic fatty liver disease (NAFLD). Hence, patients with ALD are more likely to develop cirrhosis than those with other liver diseases (Devarbhavi et al., 2023). Excessive alcohol consumption leads to abnormal accumulation of fat in the liver, and 20%–35% of such patients develop progressive liver disease, progressing from simple steatosis to hepatitis and even cirrhosis and hepatocellular carcinoma (HCC) (Poynard et al., 2003). In the past, the primary preventive measure for ALD has been complete abstinence from alcohol, and its treatment is based on glucocorticosteroids. Still, approximately 40% of patients do not respond to glucocorticosteroids (Lucey et al., 2009), whereas those who respond and use these drugs are prone to adverse reactions. Furthermore, the use of glucocorticosteroids in patients with active infection, gastrointestinal bleeding, chronic hepatitis B virus infection, or hepatic and renal syndrome is contraindicated (Depew et al., 1980; Singh et al., 2017). For patients with alcohol addiction, a step-by-step approach may be necessary; appropriate prevention and treatment at the early stage of drinking, and the use of food plants used as medicines (FPUM) to prevent ALD is an effective approach.

In traditional Chinese medicine (TCM) theory, drugs and food could have the same origin or nature, referred to as the medicine–food homology (MFH). The origin of MFH is first reflected in the allusion to “Shennong’s tasting of a hundred herbs,” during which there was no clear boundary between medicines and foods. In TCM, the concept of using medicine and food together has existed for thousands of years, and it has been widely used in various treatment and healthcare approaches of TCM (Xiang et al., 2024). As early as the *Yellow Emperor’s Classic of Internal Medicine*, the *Thousand Golden Medicinal Formulas*, and other Chinese medical textbooks, the idea that medicine and food can be used in conjunction with one another to improve human health has been embodied in FPUM. An organic combination of food therapy, medicinal diet, and health maintenance in TCM has been widely researched and applied in preventing insomnia (Ma et al., 2024), ALD (Qu et al., 2022), aging (Gao et al., 2017), and inflammation and oxidative stresses (Zhu et al., 2022; Xiong et al., 2025), which play a vital role in liver protection. FPUM demonstrates unique advantages due to their long-term consumption history ensuring both safety and pharmacological activity. The complexity of natural matrices allows multi-component synergism to mitigate toxicity risks associated with single compounds, whereas lipophilic constituents combined with plant matrix-derived bioavailability enhancers optimize pharmacokinetic profiles. Compared to synthetic drugs, their multi-target modulatory properties show superior adaptability for chronic disease management, with holistic effects from coexisting nutritional-functional components like polysaccharides and phenolics, circumventing drug resistance issues. Furthermore, botanical resources offer sustainable production advantages, where cost-effective cultivation and traditional application wisdom provide reliable translation pathways for modern drug development, particularly demonstrating irreplaceable value in environmental compatibility and social acceptability.

MFH encompass both botanical and zoological sources. In this review, we will comprehensively examine the research progress in FPUM for the prevention and treatment of ALD. Numerous studies have validated their hepatoprotective functions, with the core philosophy of alleviating alcohol-induced liver damage rooted in the TCM theory. This review first elucidates the pathogenesis of ALD, including alcohol metabolic pathways and pathogenic factors, followed by systematic analysis of the mechanisms of promising botanical drugs (*Panax ginseng* C.A. Mey., *Hovenia dulcis* Thunnb., *Pueraria montana* (Lour.) Merr., and *Hippophae rhamnoides* L. (family *Elaeagnaceae*)) in ALD management. These botanicals demonstrate multidimensional therapeutic effects: activating alcohol metabolism-associated enzymes, maintaining mitochondrial homeostasis, mitigating oxidative stress injury from alcohol consumption, and regulating cytokine networks and gut microbial ecology.

## 2 Pathophysiology of ALD

### 2.1 ALD modeling

Validation of successful ALD model establishment necessitates multidimensional pathophysiological evaluation encompassing histopathological alterations, biochemical abnormalities, and molecular biomarker fluctuations, as evidenced by hepatic steatosis (steatotic vacuoles occupying ≥30% area via hematoxylin and eosin (H&E) and Oil Red O staining), inflammatory infiltration (neutrophil/lymphocyte aggregates) with hepatocyte ballooning degeneration, and advanced stages of fibrotic septa caused by excessive collagen deposition (Masson’s trichrome/Sirius red staining); ethanol-induced serum alanine transaminase/aspartate aminotransferase (ALT/AST) elevation and dyslipidemia (triglyceride/total cholesterol↑, TG/TC); and hepatic oxidative stress imbalance (reactive oxygen species/malondialdehyde↑, ROS/MDA), glutathione/superoxide dismutase/catalase↓, GSH/SOD/CAT), and ≥3-fold upregulation of proinflammatory cytokines (tumor necrosis factor-α (TNF-α)/interleukin-6 (IL-6)/interleukin-1β (IL-1β)) in serum/liver tissue. Models must demonstrate statistically significant deviations across all parameters with histopathological progression aligning with ALD staging criteria (steatosis → steatohepatitis → fibrosis), validated through standardized ethanol exposure protocols (Lieber-DeCarli chronic model: 4–8 weeks ethanol-liquid diet; Gao binge model: cyclic ethanol gavage) (Seitz et al., 2018; Hong et al., 2024).

### 2.2 Alcohol metabolism

**FIGURE 1:**
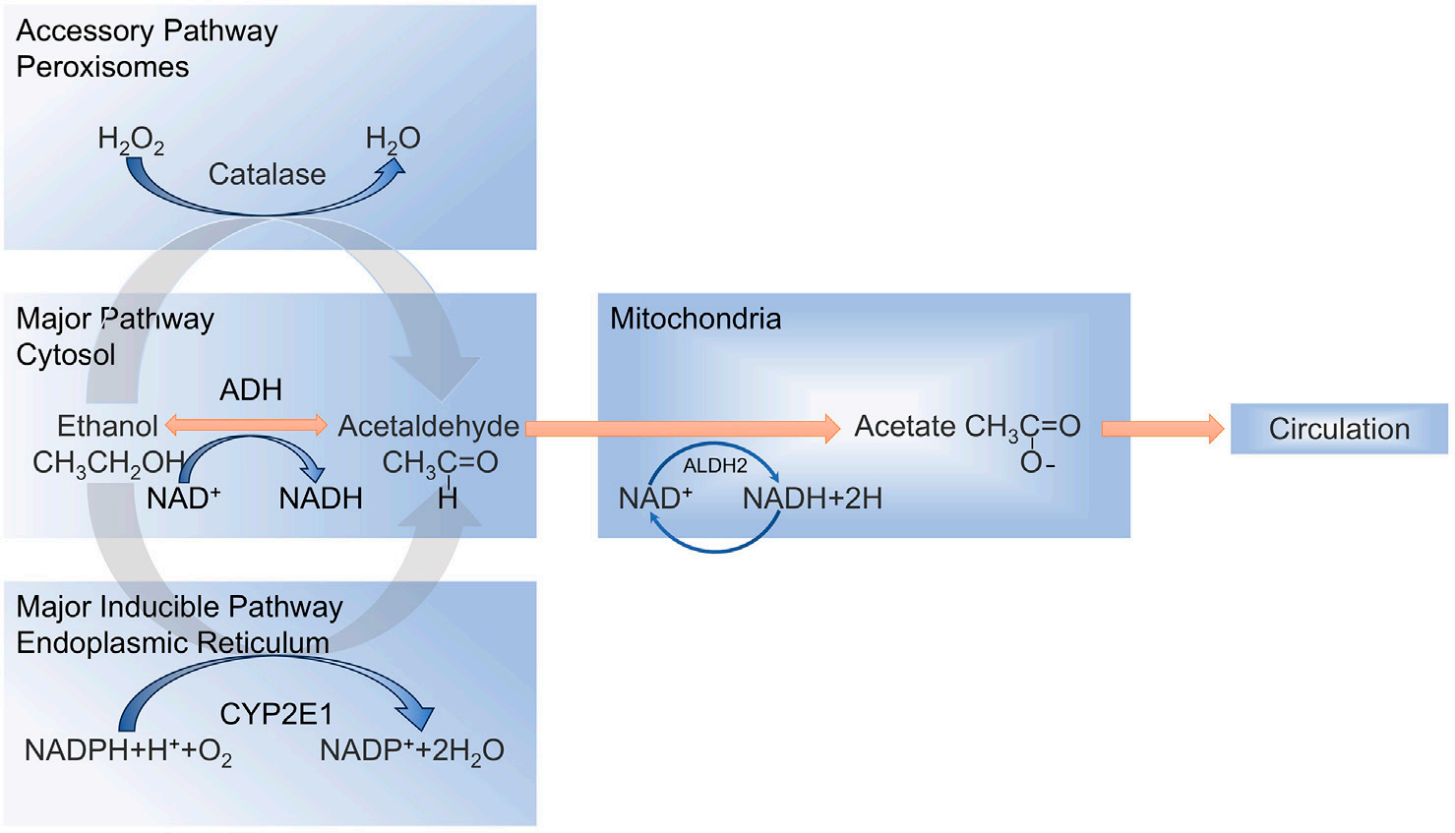
Metabolic processes involved in the oxidative conversion of alcohol.

Alcohol is oxidatively metabolized to acetaldehyde and acetic acid, primarily in the hepatocytes (Zima, 1993), and the oxidative conversion of alcohol involves three main metabolic processes (Figure 1) (Lieber et al., 1975; Guo and Ren, 2010; Cederbaum, 2012), Ethanol is mainly metabolized by the enzyme alcohol dehydrogenase (ADH) in the liver cells to produce acetaldehyde. Alternatively, ethanol is oxidized to acetaldehyde via the endoplasmic reticulum (ER) microsomal ethanol oxidation system (MEOS) through cytochrome P450 2E1 (CYP2E1), which is a major inducible oxidoreductase. The rest is oxidized by CAT using hydrogen peroxide (H_2_O_2_) into acetaldehyde and water (Oshino et al., 1973; Thurman et al., 1975; Quertemont, 2004). Excessive alcohol consumption decreases ADH activity and increases CYP2E1 activity in microsomes (Yang et al., 2012), leading to MEOS overactivity and the production of large quantities of acetaldehyde and ROS (Lu and Cederbaum, 2008; 2018; Paquot, 2019). The generated ROS disrupt the homeostatic environment within the liver, leading to a variety of hepatic responses, such as oxidative stress and inflammatory injury (Nieto et al., 2003; Dukić et al., 2023). Mechanistically, CYP2E1 induction facilitates mitochondrial shuttling of reduced nicotinamide adenine dinucleotide (NADH), amplifying ROS overproduction and recruiting immune cells that exacerbate hepatic inflammation and fibrotic progression (Hyun et al., 2021; Michalak et al., 2021).

### 2.3 Pathogenesis of ALD

The pathogenesis of ALD involves several complex links. During the oxidative metabolism of alcohol, harmful effects of alcohol metabolites, effects of endotoxins, inflammatory mediators, and cytokines, oxidative stress, autophagy, disorders of lipid metabolism, various cell death patterns, and gut microbiota dysbiosis, among others are all important causative factors (Neuman et al., 2015; Seitz et al., 2018; Slevin et al., 2020; Dukić et al., 2023; Kharbanda et al., 2023; Seitz et al., 2023; Wu X. et al., 2023).

#### 2.3.1 Harmful effects of alcohol metabolites

Acetaldehyde, a highly toxic ethanol metabolite, promotes hepatic injury through multiple mechanisms: it elevates transforming growth factor β (TGF-β) levels, thereby activating hepatic stellate cells (HSCs) and driving fibrogenesis (Jarnagin et al., 1994; Setshedi et al., 2010; Ceni et al., 2014). Through covalent binding with proteins, acetaldehyde forms acetaldehyde-protein adducts (APAs) that compromise protease activity, deplete GSH, and induce lipid peroxidation, ultimately triggering hepatocyte apoptosis. The metabolic cascade continues as acetaldehyde dehydrogenase (ALDH) oxidizes acetaldehyde to acetate, which enters the tricarboxylic acid cycle for the final conversion to carbon dioxide and water (Han et al., 2022; Wu X. et al., 2023). Genetic polymorphisms in ALDH2 exacerbate acetaldehyde accumulation, significantly increasing HCC risk (Gao et al., 2019; Wang Q. et al., 2021; Wu Y. C. et al., 2023). The above-mentioned changes collectively can lead to alcohol-induced hepatic damage.

#### 2.3.2 Disorders of lipid metabolism due to ethanol metabolism

Alcoholic fatty liver (AFL) represents a critical transitional phase in ALD progression. Ethanol disrupts hepatic lipid homeostasis through tripartite metabolic dysregulation: suppression of mitochondrial fatty acid β-oxidation via reduced nicotinamide adenine dinucleotide/NADH (NAD^+^/NADH) ratios (Masia et al., 2018), transcriptional activation of sterol regulatory element-binding protein 1c (SREBP1c) to potentiate lipogenesis, and inhibition of peroxisome proliferator-activated receptor-alpha (PPARα)-mediated lipid catabolic pathways (Hu M. et al., 2022). These concerted disturbances overwhelm physiological regulatory mechanisms of the hepatic fatty acid pool—composed of glycerol and free fatty acids—culminating in pathological lipid accumulation and steatosis (Zhou et al., 1998), a hallmark of AFL pathogenesis.

#### 2.3.3 Alcohol-induced oxidative stress

Alcohol-induced hepatic oxidative stress originates from mitochondrial ROS overproduction, which destabilizes mitochondrial DNA (mtDNA) (García-Ruiz and Fernández-Checa, 2018; Yang et al., 2019; LeFort et al., 2024).and impairs antioxidative defenses by suppressing SOD, CAT, and glutathione peroxidase (GSH-Px) activity (García-Ruiz and Fernández-Checa, 2018). Ethanol concurrently depletes GSH through adduct formation with acetaldehyde and excessive ROS neutralization. Depleted antioxidant reserves permit hydroxyl radicals to trigger lipid peroxidation (evidenced by elevated MDA levels) and DNA damage, promoting apoptosis/fibrosis (Dukić et al., 2023). ROS activate nuclear factor-κB (NF-κB), driving release of proinflammatory cytokines (IL-1, IL-6, TNF-α), which exacerbates oxidative injury, whereas hepatic hypoxia and inflammatory cell-derived ROS amplify this self-perpetuating cycle. Acute alcohol intake generates ROS through activation of the CYP2E1 pathway, and treatment targeting acute alcohol intake induces mitochondrial autophagy mediated by the putative kinase1/Parkin/microtubule-associated protein light chain 3 (PINK1/Parkin/LC3) pathway that removes damaged mitochondria, which in turn inhibits oxidative stress and hepatocyte injury (Samuvel et al., 2022). However, chronic alcohol intake inhibits autophagy, increases mTOR activity, and inhibits the hexokinase 2 (HK2)-PINK1/Parkin pathway, exacerbating mitochondrial dysfunction and hepatocyte senescence (Salete-Granado et al., 2023).

#### 2.3.4 Alcohol metabolism-induced cytokine production

Ethanol intake induces the production of multiple inflammatory cytokines and chemokines (Ambade et al., 2019). Clinical studies demonstrate significantly elevated levels of IL-1β, C–C motif chemokine ligand 2 (CCL2), monocyte chemoattractant protein 1 (MCP-1), and macrophage migration inhibitory factor in the liver and blood of patients with ALD (Bernhagen et al., 2007; Ambade et al., 2019; Ma et al., 2020). Ethanol-derived ROS stimulate hepatic Kupffer cells to generate proinflammatory cytokines, activating immune cells and triggering chemokine-mediated inflammatory cascade (Brenner et al., 2013). Lipopolysaccharide (LPS), an endotoxin from gut-derived Gram-negative bacteria, serves as a critical pathogenic mediator in alcoholic steatohepatitis. LPS activates Toll-like receptor 4/cluster of differentiation 14 (TLR4/CD14) via myeloid differentiation primary response 88 (MyD88)-dependent or independent mitogen-activated protein kinases (MAPK) signaling pathways, inducing downstream targets including activating protein-1 (AP-1) and NF-κB (Chang et al., 1997; Bode et al., 2012; Płóciennikowska et al., 2015). This process promotes inflammasome activation, upregulates inflammatory cytokines (TNF-α, IL-1β, IL-6), and triggers ER stress-mediated hepatocyte death (Lebeaupin et al., 2015). TLR4-driven M1 polarization of Kupffer cells amplifies proinflammatory cytokine/chemokine release, while ROS synergistically exacerbate hepatic steatosis and apoptosis, collectively accelerating ALD progression and fibrogenesis (Yang et al., 2019).

The complement system exhibits dual regulatory roles in ALD pathogenesis: the classical pathway (CP) aggravates inflammation through C1q-mediated apoptotic hepatocyte recognition and cytokine release (Cohen et al., 2010), whereas the alternative pathway (AP) exerts hepatoprotective effects via C3 signaling modulation. Ethanol exposure enhances C3 hydrolysis, and complement receptor 2 (CR2)-Crry-mediated inhibition of C3 activation ameliorates hepatic steatosis (Zhong et al., 2019). C3-deficient mice show resistance to ethanol-induced liver injury (Pritchard et al., 2007). Clinically, elevated plasma C4b, C5, and sC5b9 levels in patients with alcohol-associated hepatitis (AH) correlate with 90-day mortality, with complement factor I (CFI) and sC5b9 serving as prognostic biomarkers (Fan et al., 2021).

#### 2.3.5 Alcohol metabolism-mediated cell death and pro-survival pathways

ALD progression involves dysregulation of multiple programmed cell death (PCD) pathways—apoptosis, necroptosis, pyroptosis, and ferroptosis—which collectively drive hepatocyte injury and inflammation through distinct molecular mechanisms.

Apoptosis in ALD is activated via extrinsic pathways (Fas ligand/death receptor 5/microRNA-21 signaling) and intrinsic pathways. M2-polarized Kupffer cells mitigate inflammation and liver damage by releasing IL-10 to promote apoptosis of proinflammatory M1 Kupffer cells (Wan et al., 2014), suggesting therapeutic potential in modulating apoptotic pathways. Necroptosis is mediated through the receptor interacting protein kinase 1/RIP3/mixed lineage kinase domain like pseudokinase (RIP1/RIP3/MLKL) axis (Wang et al., 2018), with ethanol upregulating RIP3 expression (Zhang D. et al., 2018) to trigger damage-associated molecular pattern (DAMP) release and hepatocyte membrane rupture (Zhou Y. et al., 2022), exacerbating sterile inflammation. Pyroptosis is activated in ALD via both canonical (NOD-like receptor thermal protein domain associated protein 3 (NLRP3)-caspase-1/Gasdermin D (GSDMD)-IL-1β/IL-18) (Liu et al., 2024) and non-canonical (LPS-caspase-4/5/11-GSDMD) pathways (Yu et al., 2021; Brahadeeswaran et al., 2023). Pharmacological inhibition of IL-1β/IL-1R1 signaling attenuates hepatic inflammation and steatosis (Petrasek et al., 2012). Ferroptosis is linked to lipid peroxidation and iron dyshomeostasis in ALD. Ethanol induces iron overload, ROS accumulation, and glutathione peroxidase 4 (GPX4) inactivation while disrupting iron metabolism via SIRT1 suppression or lipin-1 upregulation (Stockwell et al., 2017; Cheng et al., 2018; Zhou et al., 2019; Zhou et al., 2020; Wu et al., 2021), aggravating dysfunction of the hepatic-gut/adipose axis. Targeting ferroptosis regulators (GPX4 reactivation and iron chelation) may offer novel therapeutic strategies for ALD.

#### 2.3.6 Autophagy and membrane transport induced by alcohol metabolism

In ALD pathogenesis, dysregulated autophagy and membrane trafficking systems critically modulate hepatocyte injury and repair. Acute ethanol exposure activates hepatocyte autophagy to clear damaged organelles (Ding W. X. et al., 2010), whereas chronic ethanol intake suppresses Rab7-mediated lysosomal function and reduces dynamin two activity (Schroeder et al., 2015; Rasineni et al., 2017), impairing lipophagy and promoting lipid accumulation. Ethanol concurrently activates mTORC1 to inhibit the nuclear translocation of transcription factor EB (TFEB), downregulating lysosomal biogenesis genes, and compromising autophagic flux (Chao et al., 2018a; Chao et al., 2018b). This autophagic impairment dynamically interacts with PCD pathways. Excessive autophagy activation promotes apoptosis via shared regulators like Beclin-1 and Bcl-2 (Fernández Á et al., 2018; Allaire et al., 2019; Li P. et al., 2020), whereas MLKL-mediated necroptosis and dysregulated endosomal trafficking enhance the production of extracellular vesicles (EVs) (Yoon et al., 2017). Chronic ethanol exposure elevates circulating EVs carrying proinflammatory mediators (miR-192, miR-30a, heat shock protein 90 (HSP90)) (Momen-Heravi et al., 2015; Saha et al., 2018), which propagate injury via HMGB1-dependent NLRP3 inflammasome activation in hepatocytes, triggering pyroptosis (Wang G. et al., 2021).

#### 2.3.7 Alcohol-induced liver regeneration

The Hippo/YAP signaling pathway regulates periportal hepatocyte gene expression during liver regeneration. In severe AH, YAP upregulation and ESRP2 downregulation induce aberrant HNF4α splicing, driving hepatocyte fetal reprogramming and ductular reactions (Bhate et al., 2015; Argemi et al., 2019; Bou Saleh et al., 2021; Wu X. et al., 2023; Isotani et al., 2025). Hepatic endothelial cells (ECs)—including periportal ECs, liver sinusoidal ECs (LSECs), and central venous ECs—orchestrate regeneration through angiocrine factors like Wnt2 and Wnt9b. However, AH induces region-specific EC alterations (Ding et al., 2010; Hu et al., 2022): periportal ECs diminish while central venous ECs expand, with WNT signaling shifting from regenerative Wnt5a to profibrotic dysregulation of the Wnt/FZD family (Kim et al., 2021). Wnt signaling exhibits stage-dependent modulation—moderate AH shows Wnt5a upregulation supporting regeneration, whereas severe AH exhibits Wnt/FZD receptor abnormalities mirroring HCC signatures (Bengochea et al., 2008; Kim et al., 2008). Central venous EC-derived Wnt9b synergizes with macrophages to activate Wnt pathways; this axis becomes dysregulated in advanced AH, impairing regeneration and promoting fibrogenesis.

**FIGURE 2:**
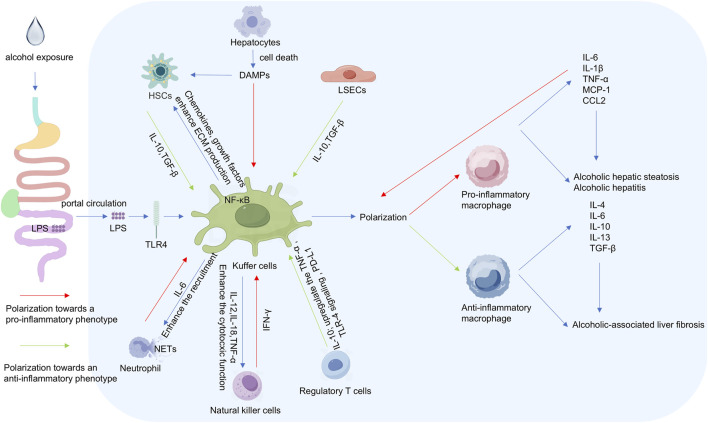
Schematic diagram of hepatocyte interaction in ALD.

LSECs and hepatic stellate cells (HSCs) drive pathological processes through dynamic interactions in ALD progression. Chronic ethanol exposure drives capillarization and defenestration of LSECs, reducing pathogen clearance and facilitating inflammatory infiltration (Xie et al., 2012). Impaired vascular endothelial growth factor (VEGF) signaling in LSECs diminishes nitric oxide synthase activity, disrupting quiescent HSC maintenance (Deleve et al., 2008). Activated HSCs express α-smooth muscle actin and secrete collagen I/III, regulated by Kupffer cell-derived TGF-β, TNF-α, and IL-1 (Bourebaba and Marycz, 2021); neutrophil-generated ROS and lipid peroxides; and complement C5a-induced chemotaxis (Martin et al., 2018; Xu et al., 2022). Ethanol downregulates miR-133 and miR-29b1, alleviating suppression of microRNA-mediated collagen synthesis (Coll et al., 2015; Zhao et al., 2019), while LSEC dysfunction further activates HSCs, exacerbating fibrosis and regenerative failure. This vicious cycle of LSEC impairment and HSC hyperactivation constitutes the core fibrotic mechanism in ALD (Figure 2).

#### 2.3.8 Organ-organ interactions in alcohol metabolism

The pathogenesis of ALD involves intricate multi-organ crosstalk, with dysregulation of the gut-liver axis and adipose-liver axis playing pivotal roles. Chronic ethanol intake induces gut microbiota dysbiosis, characterized by reduced fungal diversity and *Candida* overgrowth (Thomas, 2017), alongside increased abundance of Gram-negative bacteria (e.g., Proteobacteria), leading to elevated production of pathogen-associated molecular patterns (PAMPs), including LPS. Ethanol and its metabolites disrupt intestinal tight junction proteins (e.g., occludin, claudin-3), enhancing intestinal permeability (Rao, 2009; Mir et al., 2016), and enabling LPS translocation via the portal circulation to activate Kupffer cell TLR4 signaling, thereby triggering proinflammatory cytokine (TNF-α, IL-1β) release and hepatocyte injury. Additionally, ethanol-altered gut microbial metabolism reduces the levels of short-chain fatty acids (SCFAs), impairing their anti-inflammatory effects and exacerbating gut barrier dysfunction (Schwenger et al., 2019).

In the adipose-liver axis, ethanol stimulates adipose tissue lipolysis, releasing excess nonesterified fatty acids (NEFAs) into the liver to promote hepatic lipid accumulation and oxidative stress (Patel et al., 2022). Concurrently, adipocyte apoptosis releases complement C1q, activating complement cascades and generating proinflammatory mediators (Kema et al., 2015) that exacerbate hepatic inflammation and fibrosis via systemic circulation. Adipokines, such as leptin and chemerin, further potentiate fibrogenesis by modulating HSC activation and immune cell infiltration (Parker et al., 2018). Collectively, ALD progression is driven by synergistic multi-organ interactions, highlighting therapeutic potential in targeting inter-organ signaling networks, including gut microbiota modulation, SCFA supplementation, and adipose-lipolysis inhibition.

## 3 Current and emerging therapeutic strategies for ALD

Conventional ALD management has long relied on abstinence, nutritional support, and pharmacological interventions like corticosteroids (Mathurin et al., 2011; Fialla et al., 2015). Alcohol cessation remains the cornerstone of therapy, effectively halting steatosis progression and delaying cirrhosis (Singh et al., 2017). Clinicians must vigilantly manage alcohol withdrawal syndrome, with benzodiazepines (e.g., chlordiazepoxide) combined with vitamin B1 supplementation recommended for mitigation (Day et al., 2004; Mayo-Smith et al., 2004). Malnutrition, prevalent in patients with ALD (Mendenhall et al., 1995), necessitates high-protein dietary regimens per the American Gastroenterological Association guidelines to reduce infections and improve survival (Plauth et al., 2006). Corticosteroids yield short-term clinical benefits in 60% of patients but lack efficacy in non-responders (Singal et al., 2011). Emerging therapies target inflammatory pathway inhibition, hepatic regeneration, and gut-liver axis restoration (Jophlin et al., 2024). Pentoxifylline, a TNF-α inhibitor, demonstrates limited efficacy in severe AH by suppressing inflammation, though long-term utility remains constrained (Singal and Shah, 2016). Chronic alcohol consumption disrupts intestinal barrier integrity, inducing gut dysbiosis and bile acid dysmetabolism (Albillos et al., 2020; Shim and Jeong, 2020). Ethanol-mediated tight junction protein degradation (e.g., occludin, claudin) increases intestinal permeability, enabling bacterial endotoxin translocation via the portal vein to activate the TLR4/NF-κB signaling in hepatic cells, thereby driving inflammatory cascade and fibrogenesis (Jiang and Schnabl, 2020). Alcohol-induced gut microbiota shifts—marked by reduced beneficial taxa and pathogenic overgrowth—exacerbate immune dysregulation through enterohepatic crosstalk. Probiotics like *Lactobacillus rhamnosus* ameliorate alcohol-induced liver injury by restoring microbial balance and intestinal barrier function (Table 1) (Vatsalya et al., 2023). Fecal microbiota transplantation (FMT), an innovative approach, transfers healthy donor microbiota to recipients, effectively reducing alcohol craving, mitigating gut leakiness, and attenuating hepatic inflammation and injury (Bajaj et al., 2021).

**TABLE 1:** Targeted-gut microbiota therapies of ALD.

Type of intervention	Treatment	Main effects	References
Probiotics	Patients with ALC received *Lactobacillus casei* Shirota	Restore neutrophil phagocytic capacity; reduce sTNFR1, sTNFR2, IL10, TLR4	Stadlbauer et al. (2008)
		Reduce the intestinal permeability, hepatic steatosis and liver injury	Bull-Otterson et al. (2013)
		Increase *Clostridum* coccoides and *Eubacterium* cylindroides	Koga et al. (2013)
	Patients with ALC received *Escherichia coli* Nissle	Increase in *Lactobacillus* and *Bifidobacterium* sp.; Reduce *Proteus hausei* and *Citrobacter* sp., *Morganella* sp. and endotoxemia; improvement of liver functions	Lata et al. (2007)
	Patients with ALC received probiotic VSL#3	Reduce MDA and 4-HNE, improved TNF-α, IL-6, IL-10, and liver function	Loguercio et al. (2005)
	Patients with AH received *Bifidobacterium bifidum* and *Lactobacillus plantarum*	Reduce AST, GGT and ALT activity, lactate dehydrogenase, and total bilirubin	Kirpich et al. (2008)
	Patients with AH received *Lactobacillus subtilis* and *Streptococcus faecium*	Reduce serum LPS level and TNF-α	Han et al. (2015b)
Prebiotics	Patients with ALC received *Rifaximin*	Increase platelet count; reduce endotoxin, IL-1, IL-6,TNF-α	Kalambokis et al. (2012)
	Patients with ALD received pectin	Restore the goblet cells’ function and increases *Bacteroides* growth and prevented liver lesions	Ferrere et al. (2017)

ALC, alcoholic liver cirrhosis; HCV, hepatitis virus C.

Current research focuses on three core strategies: mitigating hepatocyte injury, suppressing inflammation, and modulating the gut-liver axis. Antioxidants like N-acetylcysteine show limited clinical efficacy, whereas mitochondrial-targeted agents, such as S-adenosylmethionine, restore glutathione levels and attenuate steatosis in preclinical models, warranting further clinical validation (Lauterburg and Velez, 1988; Moreno et al., 2010; Nguyen-Khac et al., 2011). Although the apoptosis signal regulating kinase-1 (ASK-1) inhibitor selonsertib combined with prednisolone failed to outperform steroids alone in a Phase II trial, the IL-22 agonist F-652 elevated hepatic regenerative markers and improved liver function in patients (Kong et al., 2013; Arab et al., 2020). Anti-inflammatory approaches remain challenging: corticosteroids offer transient benefits along with infection risks, while anti-TNF agents (infliximab, etanercept) increase mortality (Naveau et al., 2004). The TLR4 antagonist HA35 demonstrates preclinical efficacy in blocking TLR4 signaling but awaits clinical translation (Saikia et al., 2017). The gut-liver axis modulation by probiotics or antibiotics (e.g., amoxicillin) yields inconsistent outcomes, whereas FMT significantly improves 90-day survival in patients with severe AH while restoring microbial homeostasis in open-label trials (Philips et al., 2017; Philips et al., 2023). Phage therapy targeting cytolysin-producing *Enterococcus faecalis* reduces hepatocyte death, offering a novel approach for precision microbiome editing (Duan et al., 2019). Future strategies demand multi-target, multimodal interventions integrating single-cell omics to decode personalized immune microenvironment, alongside therapies targeting PCD pathways, autophagy-lysosomal function, and microbial metabolites to overcome therapeutic bottlenecks (Ming et al., 2024).

With an urgent need for safer and more effective ALD treatments, FPUM emerges as a historically validated therapeutic avenue. Accumulating evidence highlights the anti-inflammatory and antioxidant properties of FPUM, with bioactive metabolites modulating oxidative stress, gut microbiota, and immune response. Further exploration of FPUM’s multi-target mechanisms—particularly its regulatory effects on PCD pathways, autophagy flux, and microbial metabolites—holds transformative potential for advancing ALD therapeutic.

## 4 Mechanism of protective effect of FPUM against ALD

FPUM extracts have occupied a place in traditional medicine for thousands of years. The lack of adequate theoretical explanations in the early days has not prevented their wide application in the treatment of various diseases. Along with the rapid changes in biochemical and pharmacological technologies, researchers have conducted comprehensive and in-depth studies on the extraction, analysis, and mechanisms of action of natural plant extracts. Given the lack of effective treatments for ALD, the use of FPUM extracts for the treatment of this disease has gradually attracted attention from all walks of life. Numerous *in vitro* and *in vivo* studies have demonstrated that many natural medicines and plant extracts not only exhibit excellent antioxidant and anti-inflammatory effects but also can regulate lipid metabolism, and these properties bring new hope for the treatment of ALD, as shown in Table 2.

**TABLE 2:** Research progress in FPUM for ALD treatment.

Active metabolite	Source	Target	References
Ginsenosides	*Panax ginseng*	Inhibits oxidative stress and suppresses fat accumulation	Han et al. (2015a)
Ginsenoside Rk2	*P*. *notoginseng*	Reduces hepatic steatosis and hepatic oxidative stress, inhibits hepatitis, restores damaged intestinal barrier	Zou et al. (2023)
Ginsenoside Rb1	*P*. *quinquefolium* L.	Alleviates hepatic steatosis, reduces lipid accumulation, and mitigates inflammatory damage of ALD	Lai et al. (2021)
Ginsenoside Rc	*P*. *ginseng*	Alleviates damage and oxidative stress of hepatocytes in ALD and regulates oxidative stress, inflammation, and lipid accumulation	Pan et al. (2022)
Ginsenoside Rk3	*P*. *ginseng* CA Meyer	Alleviates oxidative stress and inhibits the expression of apoptotic proteins in the liver	Qu et al. (2019)
Salvianolic acid A	*Salvia miltiorrhiza* Bunge	Restores the activities of major ethanol-metabolizing enzymes and oxidative stress function in the liver	Shi et al. (2018a), Shi et al. (2018b)
Salvianolic acid B	*Radix Salvia miltiorrhiza*	Reduces hepatitis and fat accumulation	Zhang et al. (2017)
Puerarin	*Pueraria montana* var. *lobata*	Inhibits hepatic lipid accumulation and inflammatory response	Hu et al. (2023)
*Puerariae lobatae* Radix flavonoids and puerarin	*Puerariae lobatae* Radix	Modulates lipid metabolism and alleviates steatosis	Liu et al. (2021)
Glabridin	*Glycyrrhiza uralensis* Fisch	Alleviates alcoholic liver injury through the p38 MAPK/Nrf2/NF-κB pathway	Wang et al. (2023a)
Glycyrrhizic acid	*Glycyrrhiza glabra* L.	Regulates oxidative stress and lipid metabolism	Huo et al. (2018)
Isoliquiritigenin	*Glycyrrhiza uralensis* Fisch	Regulates miR-23a-3p/PGC-1α-mediated lipid metabolism *in vivo* and *in vitro*	Zhang et al. (2020)
Curcumin	*Curcuma longa*	Reverses damage to antioxidant system, antioxidant and anti-inflammatory, regulates lipid deposition in hepatocytes, alleviates ALD	Nanji et al. (2003), Rong et al. (2012), Lu et al. (2015), Subramaniyan et al. (2023)
*Echinacea purpurea* polysaccharide	*Echinacea purpurea*	Increases the abundances of *Muribaculaceae*, *Lactobacillus*, and *Bacteroides*, and decreases the abundances of *Escherichia coli*, *Shigella*, and *Enterococcus*	Jiang et al. (2022)
Berberine	*Coptis chinensis*	Increases the abundances of *Terrisporobacter* and *Helicobacter*, and decreases the abundances of *Pseudoflavonifractor*, *Alistipes*, *Ruminiclostridium*, and *Lachnoclostridium*	Hsiang et al. (2005), Li et al. (2020b)
Dihydromyricetin	*Hovenia dulcis*	Reduces proinflammatory cytokine levels and increases lipid phagocytosis activity, thereby enhancing lipid scavenging and inhibiting oxidative stress and cellular damage. Alleviates alcohol-induced elevated levels of inflammatory cytokines, regulates p62 and autophagy crosstalk via the Keap-1/Nrf2 pathway, and attenuates hepatic steatosis and inflammatory responses. Improves hepatic bioenergetics, metabolic signaling, and mitochondrial viability	Qiu et al. (2017), Silva et al. (2021), Janilkarn-Urena et al. (2023), Wang et al. (2023b)
Magnolol	*Magnolia officinalis*	Activates the PI3K/Nrf2/PPARγ signaling pathway and inhibits NLRP3 inflammasomes	Liu et al. (2019)

**FIGURE 3:**
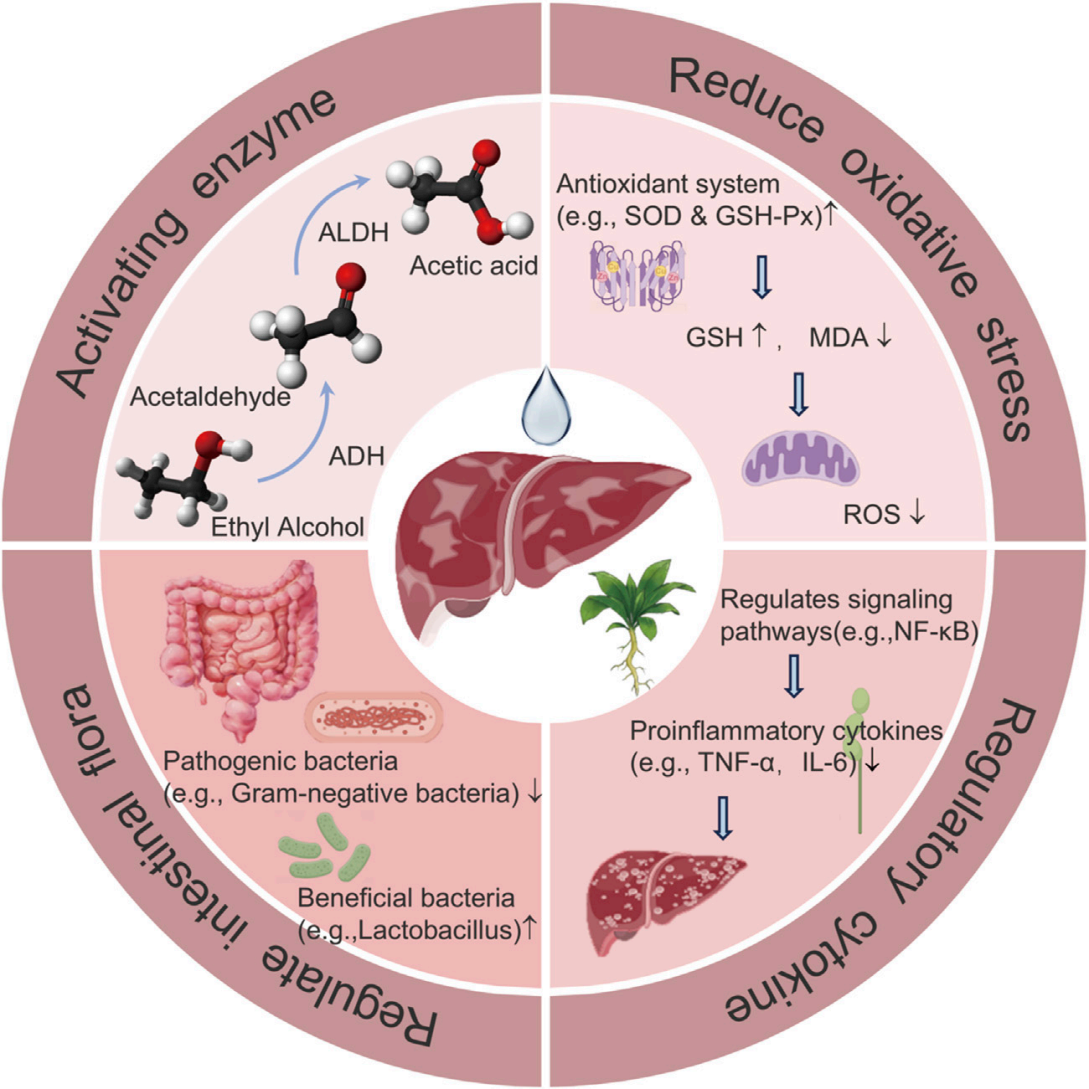
The working principle of FPUM in the treatment of ALD.

With evidence of their effectiveness and safety, FPUM have brought new hope for the treatment of ALD. First, they can speed up the breakdown and removal of alcohol from the body and reduce buildup of harmful substances by activating enzymes such as ADH and ALDH. Second, they can preserve the structural and functional stability of mitochondria, guarantee the energy supply and regular metabolism of liver cells, lessen the oxidative stress damage induced by alcohol consumption, and shield the cells from attack by free radicals. Third, FPUM can reduce the inflammatory response of the liver by controlling the proinflammatory and anti-inflammatory cytokines. Fourth, FPUM can control the composition of the intestinal flora, enhance intestinal microecology, and stabilize the intestinal-liver axis, all of which can reduce liver damage and aid in disease prevention, treatment, and recovery (Figure 3).

### 4.1 Mechanism of action of ginseng in the treatment of ALD

*Panax ginseng* C.A. Mey. is a traditional Chinese medicine widely used worldwide, and has a sweet and slightly bitter taste. It exhibits various activities beneficial for the human body such as tonifying the body’s vital energy, tonifying the spleen and lungs, generating fluids and nourishing the blood, and calming the spirit and benefiting the intellect. Therefore, research on its active metabolites and pharmacological mechanisms has attracted much attention. *Panax ginseng* C.A. Mey., *Panax quinquefolius* L., and *Panax notoginseng* (Burk) F.H. Chen (commonly abbreviated as *P. ginseng*, *P. quinquefolius*, and *P. notoginseng*), are most widely used among all species. They all contain ginsenosides as the major bioactive substances, with more than 150 monomer active metabolites (Mohanan et al., 2018; Ye et al., 2023). Few studies have shown that some ginsenosides have a wide range of therapeutic potential, such as antidiabetic, antitumor, and inhibition of NAFLD (Deng et al., 2017; Wei et al., 2018); moreover, ginsenosides attenuate damage caused by ALD (Pan et al., 2022; Yang et al., 2023). Because research on ginseng has been carried out by many mature studies, the primary bioactive metabolites ginsenosides have mature standardized products, and the clinical oral dosage is approximately 150–225 mg/d [Take Dandong Medical Creation’s Ginseng Stem and Leaf Total Saponin Tablets as an example, the implementation of the standard WS-10001-(HD-1221)-2002, the National Pharmaceutical License H21023720]. The daily dosage of approximately 30–100 mg/d is recommended for adult healthcare.

Ginsenoside Rb1 (GRb1) significantly reduces alcohol-induced lipid deposition by inhibiting hepatocyte steatosis. In terms of oxidative stress regulation, GRb1 attenuates mitochondrial dysfunction by reversing alcohol-induced ROS accumulation, partially restoring GSH levels, and decreasing MDA levels. In addition, GRb1 significantly reduces the expression of the proinflammatory factors TNF-α and IL-1β by inhibiting neutrophil infiltration into the liver parenchyma and down-regulating the NF-κB pathway. Mechanistically, the antioxidant effect of GRb1 was associated with the regulation of intracellular GSH metabolism and inhibition of ROS generation, and its anti-inflammatory effect was realized by blocking nuclear translocation of NF-κB and downstream inflammatory mediator release. Taken together, GRb1 exerts its therapeutic effects against ALD by synergistically regulating lipid metabolism disorders, oxidative stress, and inflammatory response (NF-κB/TNF-α) interaction (Lai et al., 2021; Guo et al., 2023).

Ginsenoside Rk2 (GRk2) significantly reduces serum AST/ALT levels, attenuates hepatic steatosis, and reduces lipid droplet accumulation in chronic-binge ethanol-fed mice by a mechanism related to the modulation of SREBP-1/AMP-activated protein kinase (AMPK) signaling through downregulation of fatty acid synthase (FASN), stearoyl-CoA desaturase1 (SCD1), and SREBP-1 expression, and upregulation of phosphorylated AMPK, which inhibit hepatic steatosis. GRk2 exerts antioxidant activity by upregulating the nuclear factor erythroid 2-related factor (Nrf2)/heme oxygenase-1 (HO-1) signaling pathway, thereby restoring GSH content and decreasing MDA level, and eventually inhibiting ethanol-induced oxidative stress injury. The anti-inflammatory mechanism of GRk2 involves inhibition of NLRP3 inflammatory vesicle activation in the liver by targeting NLRP3, decreasing the levels of the proinflammatory factors IL-1β and TNF-α, and reducing macrophage infiltration via inhibition of the TLR4/NF-κB pathway. Meanwhile, GRk2 upregulates regenerating islet-derived (REG)-3 lectins (REG3β/γ) and mucin protein gene (MUC2) expression by restoring the function of intestinal NLRP6 inflammatory vesicles and expression of zonula occludens-1 (ZO-1), claudin-2 (a mediator of the leaky gut barrier), and tight junction proteins (ZO-1, claudin-4), and reducing the leakage of intestinal LPS into the liver (3.74-fold decrease in intrahepatic LPS levels), further alleviating enterogenic inflammation (Zou et al., 2023). In addition, GRk2 increases fecal taurine content, activates the NLRP6/IL-18 signaling axis, regulates intestinal flora metabolism, and maintains intestinal barrier integrity. This study demonstrates for the first time that GRk2 synergistically regulates the dual targets of NLRP3/NLRP6 through the “liver-intestinal axis,” providing a new strategy for the treatment of ALD (Zou et al., 2023).

Ginsenoside Rk3 (GRk3) inhibits the expression of CYP2E1, a key enzyme in ethanol metabolism, through the inhibition of CYP2E1 expression, significantly elevates SOD and GSH activities and reduces MDA levels in the liver tissues, thereby attenuating oxidative stress injury. GRk3 effectively alleviates ethanol-induced inflammatory response by inhibiting the NF-κB signaling pathway and downregulating the mRNA and protein expression of TNF-α, IL-6, and IL-1β. In addition, GRk3 inhibits the pro-apoptotic protein BCL-2-associated X (Bax) protein and the apoptosis-inducing factors caspase-3, caspase-8, caspase-9, and poly ADP-ribose polymerase (PARP) by upregulating the expression of the anti-apoptotic protein B-cell lymphoma-2 (Bcl-2), and therefore, significantly reduces hepatocyte apoptosis. The study confirmed that Rk3 intervention significantly reduced serum ALT and AST levels and improved hepatic histopathological features, indicating that it protects liver function through multi-target synergistic effects (Qu et al., 2019).

Meanwhile, *P*. *notoginseng* saponins and Korean Red Ginseng extract (RGE) have been reported to ameliorate alcohol-induced liver injury by modulating specific signaling pathways; for example, *P*. *notoginseng* saponins exert protective effects by decreasing hepatic steatosis and oxidative stress, whereas RGE and its ginseng saponins alleviate alcohol-induced hepatic injury by activating the AMPK/SIRT1 pathway that attenuates alcohol-induced hepatotoxicity and steatosis (Han J. Y. et al., 2015). Another mechanistic study showed that ginsenoside Rg1 attenuates alcohol-induced liver injury by inhibiting excessive inflammation and hepatocyte apoptosis mediated by the NF-κB pathway (Li et al., 2018). In addition, root and leaf extracts of *P*. *notoginseng* showed protective effects against ALD, and the mechanism of action is closely related to ginsenoside (Ding et al., 2015).

In conclusion, ginsenoside and its saponin metabolites show multi-target effects in ALD treatment, and future studies need to further elucidate their effects on the hepatic-intestinal axis inflammatory signaling pathways (e.g., NLRP3/NLRP6) and metabolism regulation to promote the translation of clinical applications.

### 4.2 Mechanism of action of *Hovenia dulcis Thunnb*. In the treatment of ALD

*Hovenia dulcis Thunnb*. (HDT) is a plant belonging to the family *Rhamnaceae*; the seeds and fruits of HDT have the effect of “detoxification of alcohol poisoning” as recorded in the “Compendium of Materia Medica” (Liang and Olsen, 2014). Its seeds are also used as a diuretic, which also has a certain effect on alcoholism (Morales et al., 2017). Modern studies have further revealed that its bioactive metabolites are effective against ALD and act through multi-target mechanisms. A total of 44 metabolites have been isolated from HDT, among which flavonoids such as dihydromyricetin (DHM) and quercetin (QR), triterpenoidal saponins, and alkaloids are the primary metabolites (Sferrazza et al., 2021; He et al., 2024). DHM, QR, and naringenin have been shown to exhibit antioxidant and anti-inflammatory properties, and are also considered as potential drugs for the treatment of ALD (Qiu et al., 2019). The actual clinical oral dosage of HDT is approximately 4.5–9 g/d [for example, Jiangxi Kangzhikang’s HDT herbal tablets], and the market is dominated by pueraria and HDT being used as daily dosage for liver protection and healthcare products.

DHM significantly enhances lipophagy as evidenced by the increased co-localization of p62/SQSTM-1, microtubule-associated protein 1 light chain 3 beta (LC3B) and perilipin 1 (PLIN-1) proteins, which promote lipid droplet (LD) degradation and free fatty acid neutralization, reducing intrahepatic lipid accumulation. DHM promotes autophagic flow by enhancing LC3-II lipidation and Beclin 1 expression, removing damaged mitochondria and lipid droplets, and ameliorating hepatic steatosis (Qiu et al., 2017; Zhang J. et al., 2018; Chen et al., 2021; Janilkarn-Urena et al., 2023). Meanwhile, DHM significantly inhibits alcohol-induced CYP2E1 overexpression to improve ethanol-induced mitochondrial dysfunction by restoring the function of mitochondrial oxidative phosphorylation, enhancing β-oxidation, and decreasing the generation of oxidative metabolites such as ROS and MDA, while restoring hepatic GSH levels (Skotnicová et al., 2020). DHM inhibits NF-κB nuclear translocation, significantly downregulates the expression of the proinflammatory factors TNF-α, IL-6, and IL-17, and alleviates alcohol-induced inflammatory response. DHM promotes p62 protein binding to Keap1 through upregulation of p62 protein, thereby triggering the autophagic degradation of Keap1; it further relieves the inhibitory effect of Keap1 on Nrf2 and induces the activation of downstream antioxidant genes (HO-1) by the nuclear translocation of Nrf2 to form a positive feedback loop of p62/Keap1/Nrf2 (Qiu et al., 2017). In addition, DHM modulates lipid metabolic pathways and reduces the risk of lipotoxicity by lowering serum low-density lipoprotein (LDL/VLDL) and free cholesterol levels, while increasing cholesteryl esters and TG stores in the body. The mechanism may involve inhibition of cholesteryl ester transfer protein (CETP) and activation of lecithin-cholesterol acyltransferase (LCAT), which promotes reverse lipid transport (Janilkarn-Urena et al., 2023). Chronic alcohol intake inhibits AMPK phosphorylation (Thr172) and Sirt-1/PGC-1α expression, leading to inactivation of PGC-1α by hyperacetylation, which in turn impairs mitochondrial function and reduces adenosine triphosphate production. DHM activates AMPK by elevating hepatic NAD + levels (43% increase in phosphorylation), restores the expression of Sirt-1 (37%) and mitochondrial Sirt-3 (31%), and promotes deacetylation of PGC-1α, thereby restoring its transcriptional co-activation function (Silva et al., 2021). In summary, DHM synergistically ameliorates alcoholic liver injury by regulating antioxidant, autophagic, and anti-inflammatory pathways, activating lipophagy, improving mitochondrial function, and inhibiting inflammatory signaling pathways, which provides a potential precision therapeutic strategy for ALD.

QR significantly reduces ethanol-induced ALT, AST and gamma-glutamyltransferase levels, decreases hepatic TG and TC accumulation, enhances the expression of the antioxidant genes HO-1 and GPx, and improves mitochondrial function to inhibit hepatic lipid droplet deposition. QR enhances the activity of hepatic autophagy through AMPK activation and promotes co-localization of LC3-II with the lipid droplet-associated protein PLIN-2, thereby accelerating lipophagy degradation. By activating AMPK, QR enhances autophagy activity of hepatocytes and promotes co-localization of LC3-II with the lipid droplet-associated protein PLIN-2, which accelerate lipophagy degradation and significantly alleviate alcohol-induced hepatic steatosis. QR ameliorates ethanol-induced hepatic steatosis through the modulation of the purinergic 2 × 7 receptor (P2X7R)-mediated PI3K/Keap1/Nrf2 oxidative stress signaling pathway (Zhao et al., 2021), inhibits ROS production and enhances antioxidant enzyme activities, effectively attenuates ethanol metabolism-induced significant upregulation of the mRNA expression of PI3K, Keap1, and Nrf2, enhances the activities of antioxidant enzymes (SOD, GSH, CAT), and reduces the content of lipid peroxidation products (MDA). In terms of inflammation regulation, QR significantly reduces the expression of the proinflammatory factors TNF-α, IL-6, and NF-κB (Markowska et al., 2024), and inhibits the Kupffer cell TLR4-mediated inflammatory cascade. QR metabolites may indirectly improve the hepatic inflammatory microenvironment by modulating the composition of the intestinal flora and decreasing endotoxin translocation (Yan et al., 2021). Studies have also shown that QR promotes lipophagy through the endoplasmic reticulum stress-sensing protein IRE1α/XBP1s (transmembrane kinase/endoribonuclease 1α/X-box binding protein 1) pathway, accelerating lipodroplet catabolism and VLDL assembly to further reduce the hepatocyte lipid load (Zhu et al., 2018). In terms of intestinal flora regulation, QR increases the abundance of beneficial bacteria such as *Akkermansia*, reduces LPS translocation, and inhibits the TLR4/NF-κB signaling pathway, thereby improving intestinal barrier function and reducing systemic inflammation (Porras et al., 2017; Shi et al., 2022). In addition, QR combined with dasatinib cleared senescent hepatocytes, reduces senescence-associated secretory phenotype (SASP)-associated release, and slows down the process of liver fibrosis (Palmer et al., 2019). These results suggest that QR ameliorates oxidative damage and inflammation in ALD through multi-target regulation of oxidative stress and lipid metabolism disorders, providing a molecular mechanism for the treatment of ALD.

Naringenin accelerates the conversion of ethanol to acetic acid and reduces the accumulation of the toxic metabolite acetaldehyde by upregulating the activities of ADH and ALDH, while inhibiting the expression of CYP2E1 and decreasing the production of ROS during ethanol metabolism (Jayaraman and Namasivayam, 2011). Naringenin upregulates the expression of GSH synthase and antioxidant enzymes (SOD, CAT, and GSH-Px) through the activation of the Nrf2 signaling pathway, restores the GSH/GSSG balance (Gopinath and Sudhandiran, 2012), and reduces MDA (a lipid peroxidation product) levels (Jayaraman et al., 2012), effectively scavenging free radicals and inhibiting lipid peroxidation. In addition, naringenin reduces the expression of the inflammatory factors TNF-α, IL-6, cyclooxygenase-2 (COX-2), and inducible nitric oxide synthase (iNOS) by inhibiting the NF-κB pathway, thereby reducing the hepatic inflammatory response. In terms of lipid metabolism, naringenin reduces hepatic inflammation by downregulating the expression of 3-hydroxy-3-methylglutaryl CoA reductase (HMGCR), acyl-coenzyme A: cholesterol acyltransferase (ACAT), and microsomal triglyceride transfer protein (MTP), inhibiting VLDL assembly, reducing intracellular cholesteryl ester and TG accumulation in hepatocytes, and ameliorating alcohol-induced steatosis (Nahmias et al., 2008). The above mechanisms act synergistically, resulting in therapeutic potential of naringenin in ALD through cell necrosis, improvement of liver function indices (TC and TG levels), and histopathological features.

Alcohol promotes SREBP-1c-mediated adipogenesis by inhibiting the AMPK and PPAR-α signaling. DHM, apigenin, and QR activate AMPK, inhibit acetyl coenzyme A carboxylase and fatty acid synthase activities, and reduce triglyceride synthesis. Chronic alcohol exposure induces hepatocyte apoptosis and ferroptosis. DHM, β-sitosterol, and naringenin reduce apoptosis by inhibiting the aspartate-specific cysteine protein hydrolase-3 (CASP3) pathway (Zhao et al., 2018; Chen et al., 2020); naringenin decreases ferritin expression in the liver (Jayaraman et al., 2012), and QR reduces intracellular destabilized iron pools by maintaining the level of free iron-mediated ⋅OH production and attenuates alcohol-induced liver injury (Li et al., 2014; Li et al., 2016). Alcohol disrupts intestinal tight junction proteins (ZO-1) and leads to dysbiosis of the intestinal flora. Lutein increases the abundances of *Bifidobacterium*, *Subdoligranulum*, and *Faecalibacterium prausnitzii* (Zhao et al., 2023). Although HDT showed significant hepatoprotective effects *in vitro* and in animal experiments, its clinical translation still faces challenges, the mechanism of multi-component synergism is still unclear, and the future studies need to determine the key pharmacodynamic substances through the spectral correlation technique.

### 4.3 Mechanism of action of *Pueraria* in the treatment of ALD

*Pueraria montana* (Lour.) Merr. is the root of the legume *Pueraria*, and has a sweet and pungent flavor; it is a classic FPUM and has beneficial effects in the human body, including relieving fever, generating fluids and quenching thirst, penetrating rashes and removing vexation, elevating yang, stopping diarrhea, as well as relieving alcoholism. Modern pharmacology has shown that *Pueraria* can reduce lipid deposition, vasodilate blood vessels, inhibit inflammation, and relieve hangover (Fang et al., 2022), which is largely consistent with its traditional uses. Puerarin is the main bioactive metabolite isolated from *Pueraria* and widely used for the treatment of cardiovascular diseases, diabetes, and liver diseases (Zhang, 2019). Clinical detoxification often involves supplementation with *Pueraria-*based products and conventional drugs for the treatment of ALD, and Pueraria products are mostly used for the prevention and relief of symptoms of intoxication and treatment of mild alcoholism. However, there is no study showing use of single-flavored *Pueraria* product for the treatment of ALD. This may be related to the fact that *Pueraria* has a slow onset of action when taken in small doses, while large doses may cause adverse reactions. Puerarin has the characteristic of “small dose, narrow treatment,” according to the 2015 edition of the Pharmacopoeia of the People’s Republic of China, the daily dosage of *Pueraria* should not be higher than 15 g, and Pueraria has no allergic reaction in small doses, but large doses of Pueraria added to other formulas will cause allergic reactions in few individuals. Therefore, patients who experience frequent allergies should not mix *Pueraria* with other TCM to treat alcoholic intoxication. The clinical oral dosage of *Pueraria* is approximately 9–12 g/d [Take Pueraria Powder from Anhui Kanghe TCM, for example, approval number YUN ypbz-0210–2014].

Puerarin reduces ROS production by inhibiting the overactivation of CYP2E1 and CYP3A in hepatic MEOS, while elevating the activities of endogenous antioxidant enzymes, such as SOD, GSH-Px, and CAT, to alleviate oxidative stress damage (Zhao et al., 2010; Chen et al., 2013; Zhao et al., 2016; Yan et al., 2021). Puerarin significantly reduces the expression of the proinflammatory factors TNF-α, IL-1β, and IL-6 by inhibiting intestinal endotoxin leakage and Kupffer cell activation, and downregulating the TLR2/4 and NF-κB signaling pathways, thus reducing the inflammatory response (Peng et al., 2013; Greatorex et al., 2023). Regarding the regulation of lipid metabolism, the glycoside enhances autophagy activity through activation of the AMPK/mTOR pathway, promotes lipophagy, and reduces hepatic accumulation of TG and TC, while inhibiting the expression of SREBP-1c to reduce lipid synthesis (Noh et al., 2011; Zhou et al., 2014). Meanwhile, its modulation of glycogen synthase kinase-3β (GSK-3β) phosphorylation blocks the NF-κB-mediated inflammatory cascade response and inhibits COX-2 and 5-lipoxygenase (5-LOX) activities to reduce leukotriene B4 (LTB4) production (Li et al., 2013; Tian et al., 2021). In addition, puerarin inhibits TGF-β1-mediated hepatic stellate cell activation and collagen deposition, reduces extracellular matrix (ECM) production, and upregulates matrix metalloproteinase (MMP-1/MMP-2) expression to reverse fibrosis (Guo et al., 2013; Lee et al., 2023), through the modulation/reversal of the TGF-β1/Smad pathway (Xu et al., 2013). In summary, the therapeutic mechanism of puerarin in ALD is mainly through the multi-pathway regulation of oxidative stress, inflammatory response, lipid metabolism, and cell death, and through the multi-targeted regulation of oxidative-antioxidant homeostasis and organelle homeostasis to produce protective effects against acute alcoholic liver injury.

### 4.4 Mechanism of action of sea buckthorn in the treatment of ALD

Sea buckthorn (*Hippophae rhamnoides* L., family *Elaeagnaceae*) is a plant containing various metabolites, including flavonoids, carotenoids, sterols, tocopherols, and lipids, which produce a variety of effects such as antimicrobial, anti-inflammatory, regulating blood pressure, antioxidant, and anticancer effects, and it is a valuable medicinal and dual-use plant (Gong et al., 2015). Sea buckthorn extract has shown protective effect against acrylamide-associated brain damage (Turan et al., 2021); it can be used to treat myocardial ischemia, and has been widely used for its antioxidant, anti-inflammatory, antimicrobial, wound healing and other dermatological properties (Pundir et al., 2021). It can also improve fat deposition, hepatic steatosis, insulin resistance, and inflammation in dietary-induced obesity (Kwon et al., 2017). The clinical oral dosage of sea buckthorn is 30–45 g/d [Sea buckthorn granules from Gansu Lan Pharmaceutical, for example, State Drug License Z62020982], while the recommended dosage of sea buckthorn syrup as a healthcare product is 20–30 mL/d [Sea buckthorn syrup drink from Jiangxi Renhang, for example, Approval Certificate No. SC10636098210527].

*H*. *rhamnoides* fermentation liquid (HRFL) alleviates alcohol-induced oxidative stress by scavenging free radicals, enhancing SOD activity, and lowering MDA levels; it also inhibits the expression of the proinflammatory factors TNF-α and IL-6, and attenuates hepatic inflammatory responses. In the regulation of lipid metabolism, HRFL significantly downregulates serum LDL-C and TG, upregulates HDL-C, and reduces hepatic lipid deposition (Ran et al., 2021), while *H*. *rhamnoides* flavonoids extract (HRFE) also improves hepatocellular steatosis by lowering the levels of serum ALT, AST, TC, and TG. In terms of intestinal microecological regulation, HRFL enhances intestinal barrier integrity by increasing the production of SCFAs such as butyric acid and acetic acid, and modulates the intestinal flora; it inhibits the proliferation of ALD-associated genera *Alistipes* and *Ruminiclostridium* and upregulates the abundance of the beneficial bacterium *Lactobacillus* to reduce the endotoxin leakage. The combination of HRFE and *H*. *rhamnoides* polysaccharide (HRP) has shown to restore the α-diversity of the intestinal flora and reverse alcohol-induced changes in the *Firmicutes*/*Bacteroidetes* ratio (F/B), with HRP specifically inhibiting the proliferation of the proinflammatory genera *Clostridiales* and *Luminiclostridium* in *Firmicutes* and increasing the abundance of S24-7 in *Bacteroidetes*. Moreover, it reduces the portal vein entry of LPS into the liver through the “gut-hepatic axis” mechanism, which inhibits the activation of Kupffer cells and release of inflammatory factors (Liu et al., 2022; Zhao et al., 2022). At the molecular level, HRFE inhibits the activation of downstream p38MAPK and p65NF-κB signaling pathways by blocking the phosphorylation of TAK1, and downregulates the mRNA and protein expression of TNF-α, TGF-β, and IL-6 (Zhao et al., 2022). HRP indirectly affects intrahepatic lipid metabolism by regulating the metabolites derived from the bacterial flora (Liu et al., 2022). In summary, sea buckthorn intervenes in ALD pathology at multiple targets through its synergistic effects of antioxidant, anti-inflammatory, lipid metabolism regulation, intestinal barrier repair, and inhibition of the TAK1/p38MAPK/NF-κB inflammatory signaling pathways.

Drugs used to treat ALD (for example, curcumin, resveratrol, and glycyrrhizic acid) exert their therapeutic effects by regulating macrophage polarization, along with inhibition of proinflammatory M1 (CD86+/iNOS+) and activation of anti-inflammatory reparative M2 (CD206+/Arg-1+) as the key mechanisms (Li et al., 2019; Zhou L. et al., 2022). IL-10, an anti-inflammatory cytokine, enhances M2 polarization through the STAT3 pathway, inhibiting the release of TNF-α and IL-6, while upregulating the expression of arginase-1 (Arg-1), which reduces nitric oxide (NO) toxicity by metabolizing arginine and promotes polyamine and proline synthesis to accelerate liver tissue repair. For example, silymarin inhibits NF-κB through PPARγ activation, reduces the M1 marker IL-1β, and induces Arg-1-mediated reversal of fibrosis (Surai et al., 2024). Tanshinone IIA attenuates ethanol-induced hepatic injury through upregulation of the IL-10/STAT6 axis driving M2 polarization (Koushki et al., 2015). Such modulations can synergistically improve the inflammatory microenvironment and oxidative stress in ALD and delay the fibrosis process.

## 5 Discussion

This study provides a systematic review of the pathogenesis of ALD and the interventional role of FPUM. The core pathological processes of ALD involve disturbances in alcohol metabolism (acetaldehyde toxicity, CYP2E1 activation), oxidative stress overload (ROS accumulation, inhibition of antioxidant enzymes), activation of inflammatory signaling (for example, the TLR4/NF-κB/NLRP3 pathways), programmed cell death (apoptosis, necrotic apoptosis, pyroptosis, and ferroptosis), and dysregulation of the gut-liver axis (LPS leakage, flora imbalance). In response to the limited effectiveness and significant side effects of traditional therapeutic approaches, FPUM demonstrate unique advantages through multi-component synergistic effects. For example, ginsenosides (GRb1, GRk3) improve lipid metabolism and oxidative stress by inhibiting CYP2E1 and regulating the AMPK/SIRT1/Nrf2 signaling pathway; DHM in HDT activates the autophagy-lipophagy pathway and restores mitochondrial function; and puerarin and seabuckthorn flavonoids mediate hepatoprotection through the antagonism of inflammatory vesicles and regulation of the diversity of intestinal flora. In addition, FPUM can meet the needs of chronic disease management through multi-targeted interventions and has shown potential to complement conventional drugs in complex aspects of liver regeneration (e.g., Hippo/YAP pathway) and fibrosis reversal (e.g., TGF-β/Smad modulation). Nevertheless, existing studies are mostly limited to animal models and *in vitro* experiments, with insufficient evidence for clinical translation, and systematic validation of their safety and efficacy is urgently needed.

Future research should focus on the following directions: (1) In-depth analysis of molecular mechanisms: Mass spectrometry should be used to identify the key active metabolites of FPUM’s multi-component synergism, combined with single-cell sequencing and organoid modeling to elucidate its specific regulatory network on hepatocyte subpopulations and nonparenchymal cells (e.g., Kupffer cells, HSC). For example, the molecular dynamics of ginsenosides in regulating NLRP3/NLRP6 inflammatory vesicles may reveal its “liver-gut axis” interaction mechanism. (2) Preclinical and clinical trial design: A standardized ALD staging animal model (e.g., the Gao binge-Ethanol model) needs to be established to evaluate the quantitative and quantitative effects of FPUM at different stages of the disease. Clinical trials should be advanced in phases: phase I focuses on evaluating toxicity and pharmacokinetics; phase II explores the synergistic effect of combination therapies (e.g., with selonsertib or IL-22) on severe alcoholic hepatitis, and detects novel biomarkers, such as serum sC5b9 and intestinal barrier markers (e.g., claudin-3, LBP); and phase III verifies the benefits of long-term intervention on fibrosis reversal and hepatocellular carcinoma prevention. (3) Potential for drug synergistic application: Combination of FPUM with existing drugs may overcome monotherapy limitations. For example, puerarin combined with glucocorticoids may reduce the latter’s dose and alleviate the risk of immunosuppression; HDT polysaccharides in combination with probiotics may enhance the protective effects of the intestinal barrier through the regulation of the flora-metabolite axis (e.g., SCFAs, bile acids). In addition, the development of nano-delivery systems (e.g., liposomal encapsulation of ginsenosides) may enhance bioavailability, and the integration of herbal remedies with modern precision medicine strategies may open up new avenues for ALD treatment.

Although FPUM show multi-target therapeutic potential, its clinical application still faces the following core challenges: (1) Dosage standardization and quality control challenges: the complexity of FPUM’s composition and geographical variability make it difficult to define the dose-effect relationship. Studies have shown that the content of Sr, Rb, and other key active metabolites in HDT varies significantly depending on the region of origin (e.g., Shanxi vs. Jiangxi) (Sr content fluctuates from 1.80 to 62.8 mg/kg), which produces inconsistency in therapeutic efficacy. In addition, most FPUM (e.g., *Pueraria*) have a narrow therapeutic window, exceeding the upper limit of dosage is prone to sensitization or liver damage, but the current Chinese Pharmacopoeia only partially limits the medicinal dosage (e.g., *Pueraria* ≤ 15 g/d), and lacks a precise quantitative standard for the active metabolite (e.g., ginsenoside Rk3). (2) Bioavailability and pharmacokinetic bottleneck: The oral bioavailability of polysaccharides and saponins in FPUM is generally low due to their large molecular weight, poor water solubility, and obvious first-pass effect. For example, single oral absorption rate of QR is less than 20%, and the metabolism of intestinal flora may change its active form. Although fat-soluble components (e.g., flavonoids) can show enhanced absorption via liposomes or nano-delivery systems (e.g., chitosan encapsulation), they still face the problem of low solubility and insufficient transmembrane transport efficiency. Animal models show that DHM has a short retention time in the liver tissue (half-life <4 h) and needs to be administered frequently to maintain efficacy, which may limit clinical translation. (3) Insufficient clinical validation and grade of evidence: most studies on FPUM intervention in ALD have focused on cell or animal models, and clinical trials are still in their infancy. For example, only a few phase I/II trials have validated the safety of ginsenosides (e.g., GRb1 alone or combined with YN-3 probiotic intervention in mild ALD), but with limited sample sizes (n < 100), shorter duration of treatment (≤12 weeks), and lack of long-term follow-up data on endpoints with hard indicators (e.g., rate of reversal of hepatic fibrosis, incidence of HCC). In addition, the synergistic combination of metabolite preparations (e.g., Pueraria - HDT ratio) has not been verified by rigorous double-blind randomized controlled trials (RCTs), and the theory of “multi-component-multi-targets” is in urgent need of translational medicine support. (4) Defects in production process and quality standards: Traditional extraction processes (e.g., water decoction, alcohol precipitation) can easily lead to the degradation or imbalance of active metabolites (e.g., saponins, polysaccharides). For example, the synergistic effect of flavonoids and polysaccharides in sea buckthorn extract is limited by the separation and purification technology, and industrial production may weaken its “overall effect”. At the same time, FPUM raw materials are at risk of heavy metal contamination. Studies show that the Al content of some botanical drugs (e.g., HDT) is as high as 17.5 mg/kg, which may exacerbate liver damage, but the existing standard of “Heavy Metal Limits for TCM” only covers few elements (e.g., Pb, Cd), and there is no safety threshold for trace elements such as Sr, Rb, and so on. (5) Insufficient multidisciplinary integration and policy support: Modernization of TCM requires the integration of pharmacology, synthetic biology, and artificial intelligence (AI) technology, but current research is scattered and lacks systematic approach. For example, although the mechanism of intestinal flora-FPUM interactions has been emphasized, there is a lack of joint analysis of flora metabolome and host epigenetics. In addition, there is a disconnect between international natural product standards (e.g., ISO 18664) and domestic registration requirements (e.g., Guidelines for Clinical Research of New Chinese Medicines), which makes it difficult to expand the chain of evidence for FPUM through international multicenter trials. In conclusion, to break through the bottleneck of FPUM in treating ALD, it is necessary to establish a whole chain research system of “composition analysis - process optimization - clinical validation - policy coordination,” and at the same time, to strengthen the integration of multi-omics technology and traditional medical experience in a manner to realize the efficient translation from laboratory to clinic.
